# A molecular and staging model predicts survival in patients with resected non-small cell lung cancer

**DOI:** 10.1186/s12885-018-4881-9

**Published:** 2018-10-11

**Authors:** Lei Liu, Minxin Shi, Zhiwei Wang, Haimin Lu, Chang Li, Yu Tao, Xiaoyan Chen, Jun Zhao

**Affiliations:** 1 Department of Thoracic and Cardiovascular Surgery, The First Affiliated Hospital of Soochow University, Medical College of Soochow University, Suzhou, Jiangsu Province, China; 2 Department of Thoracic Surgery, The Affiliated Tumor Hospital of Nantong University, Nantong, Jiangsu Province, China; 3 Department of Breast, International Peace Maternity and Child Health Hospital, Shanghai Jiao Tong University, Shanghai, China; 4 Department of Pathology, The Affiliated Tumor Hospital of Nantong University, Nantong, Jiangsu Province China; 5 Department of Pathology, Shanghai Ruijin Hospital affiliated to Shanghai Jiao Tong University School of Medicine, Shanghai, China

**Keywords:** Non-small cell lung cancer, Survival, TPX2, MMP12

## Abstract

**Background:**

The current TNM staging system is far from perfect in predicting the survival of individual non-small cell lung cancer (NSCLC) patients. In this study, we aim to combine clinical variables and molecular biomarkers to develop a prognostic model for patients with NSCLC.

**Methods:**

Candidate molecular biomarkers were extracted from the Gene Expression Omnibus (GEO), and Cox regression analysis was performed to determine significant prognostic factors. The survival prediction model was constructed based on multivariable Cox regression analysis in a cohort of 152 NSCLC patients. The predictive performance of the model was assessed by the Area under the Receiver Operating Characteristic Curve (AUC) and Kaplan–Meier survival analysis.

**Results:**

The survival prediction model consisting of two genes (TPX2 and MMP12) and two clinicopathological factors (tumor stage and grade) was developed. The patients could be divided into either high-risk group or low-risk group. Both disease-free survival and overall survival were significantly different among the diverse groups (*P* < 0.05). The AUC of the prognostic model was higher than that of the TNM staging system for predicting survival.

**Conclusions:**

We developed a novel prognostic model which can accurately predict outcomes for patients with NSCLC after surgery.

**Electronic supplementary material:**

The online version of this article (10.1186/s12885-018-4881-9) contains supplementary material, which is available to authorized users.

## Background

Lung cancer is one of the most common cancers and its death rate ranks among the top of the all tumors worldwide [1]. Almost 80% of all lung cancer deaths were non-small cell lung cancer (NSCLC). The overall survival of NSCLC remains poor, although there are tremendous developments of treatments [2]. Tumor-node-metastasis (TNM) system is widely used to estimate the outcome of patients with NSCLC in current clinical practice. However, a completely different prognosis may occur in patients with the same TNM stage [3] .Other traditional clinicopathologic factors including age, sex, pathological type and tumor grade have been reported to correlate to the prognosis of NSCLC [4]. Moreover, it is also believed that tremendous heterogeneity between patients exists in the biology underlying NSCLC. Therefore, the ideal staging system would join the biology and molecular features of each individual tumor, and correlate prognosis with patient-specific tumor biomarkers.

Recently, the microarray technique which can investigate gene expression systematically enabled us to visualize gene expression profiles in human cancers. People used more and more gene expression to predict the prognosis in different type of tumors [5–8]. For instance, Cao et al. proposed a molecular model based on three genes which could accurately predict the survival of patients of esophageal squamous cell carcinoma [9]. A subnetwork constructed by five signatures could be applied to divide the colorectal cancer patients into high or low risk group [10]. Differences in survival were found between lung cancer patients with and without DNA alterations in genes encoding the metabolism proteome [11]. A predictive 7-gene assay and prognostic protein biomarkers were established for improving NSCLC treatment. These findings could divide cancer patients into high or low risk group [12]. However, these authors did not use clinical and pathological information in their studies. In the present study, we investigated the prognostic value of the traditional clinicopathological factors and selected protein expression. We aimed to build a novel predictive model, which would be capable of predicting outcomes of NSCLC patients after surgery.

## Methods

### Microarray data analysis

We obtained the microarray data from the publicly available Gene Expression Omnibus databases (GSE 31552 and GSE 18842) in this study. R/BioConductor was used for preprocessing the microarray text data from BeadStudio. The expression level of each gene was transformed into a log 2 base before further analysis. Then, we calculated the log2 fold change (FC) of each probe on the array within each tissue pair. The differential expression between tumor tissue and matched normal mucosa was tested using rank product test. The differential expression was declared significant if the adjusted *p*-value, i.e. the FDR q-value, was less than 0.05.

### Study patients

A total of 152 NSCLC patients undergoing curative resection at Nantong tumor hospital between 2011 and 2012 were enrolled in the study. We followed up all the patients using a standard protocol after being discharged from the hospital. The follow-up was carried out until the end of July 2017. The disease free survival was defined as the interval from the date of surgery to the date of local or regional disease recurrence, distant metastasis, or to the last follow-up date. The overall survival was calculated from the time of surgery to the time of death for any cause, or to the time of last follow-up. All the cases were diagnosed as NSCLC by the pathologists. We retrieved patients’ clinical information, sex, age, pathological type, and TNM stage (using the 8th UICC TNM Staging System of NSCLC) from their medical records. The clinicopathological characteristics for the patients were listed in Table 1. This study was approved by the institutional review boards of Nantong tumor hospital. A written informed consent was obtained from each patient.

**Table 1:** Clinicopathological factors of patients in the study

Variables	No.	Percent
Age (years)		
< 60	63	41.4
≥ 60	89	58.6
Gender		
Male	83	54.6
Female	69	45.4-
Smoking status		
Never-smoker	64	42.1
Ever-smoker	88	57.9
TNM stage		
I	53	34.9
II	51	33.6
III	48	31,5
Grade		
Well-differentiated	32	21.1
Moderately-differentiated	66	43.4
Poorly-differentiated	54	35.5
Histology		
Squamous cell carcinoma	39	25.7
Adenocarcinoma	113	74.3
Postoperative adjuvant therapy		
Yes	84	55.3
No	68	44.7

### Real-time quantitative reverse transcription PCR

A total of 1 μg mRNA from each sample was reversely transcribed to single stranded cDNA, using an Advantage RT for PCR kit (Clontech). We used SYBR Green reagent (Applied Biosystems, CA, USA) to analyze mRNA expression for qRT-PCR. The PCR reactions were performed at 95 °C for five minutes, then a 3-step cycle procedure was performed (denaturation at 95 °C for 10 s, annealing at 60 °C for 20 s, and elongation at 72 °C for 40 s) for 35 cycles, with a final extension at 72 °C for 10 min. GAPDH was served as the endogenous control. The primers used for qRT-PCR analysis were listed in Additional file 2: Table S1. The comparative Ct (threshold cycle) method was used to calculate the relative changes in gene expression.

### Immunohistochemical analysis

We cut the formalin-fixed, paraffin-embedded lung cancer tissues into 4-μm sections and then mounted them on slides. We blocked endogenous peroxidase by incubating the sections in a 0.3% solution of hydrogen peroxide (in PBS) for 10 min. Then we heated the sections for 10 min for antigen retrieval at 100 °C in 10 mM citrate buffer (pH 6.0). Sections were incubated overnight at 4 °C with mouse anti-TPX2 (1:200 dilution, ab32795, Abcam, Cambridge, UK) and rabbit anti-MMP12 (1:50 dilution, SC-30072, Santa Cruz, Texas, USA) antibodies. Then, in order to develop peroxidase activity for visualizing the antibody-drochloride complex, the slides were reacted with Novolink polymer followed by DAB chromogen solution. We counterstained all the Slides with haematoxylin. Two pathologists blinded to patients’ background independently scored the staining of TPX2 and MMP12. We calculated the sum of the percentage and intensity of positively stained invasive tumor cells to perform immunostaining scoring for each sample. The intensity of positive cells was grouped by four grades: 0, 1, 2 and 3 for negative staining, weak staining, moderate staining and strong staining, respectively. The final staining score was calculated by the following method:score = intensity score x percentage score ((1*%1+) + (2*%2+) + (3*%3+)), which ranged from 0 to 300. The cut off score for protein expression was determined by X-tile [13]. Positive staining was interpreted as score > 40 for TPX2 and > 50 for MMP12, respectively.

### Statistical analysis

Paired t test was used to compare the mRNA expression in cancer and matched adjacent normal tissues. Cox regression analysis was used to determine the prognostic significance of clinical and pathologic features. The significant prognostic factors in Cox regression analysis were chosen to establish the survival prediction model. To construct a predictive model, each of the selected prognostic factors was analyzed using a multivariable Cox regression model, with DFS or OS as the dependent variable and other clinical information as the covariables. A risk score was then computed as follows: Y=$\sum \limits_{i=1}^n$(ki*xi),where Y is the risk score, N is the number of prognostic factor, xi is the value of prognostic factor, and ki is the estimated regression coefficient of prognostic factor in the multivariable Cox regression analysis. The median risk score was considered as a cut off value. The Kaplan-Meier method was used to estimate the survival of patients in different groups, and the two side log-rank test was applied to determine the statistical significance. Receiver operating characteristic (ROC) curve was used to compare the sensitivity and specificity of the prognostic parameters. All data analysis was performed by using SPSS 15.0 software. The *P* value less than 0.05 were considered as significant.

## Results

### Candidate gene selection

In GSE18842 dataset, 46 NSCLC samples were included. There were 14 adenocarcinomas and 32 squamous-cell carcinomas cases, respectively; 45 of them were paired with their corresponding nontumor sample. A total of 30 pairs NSCLC and non-tumor samples (10 pairs squamous-cell carcinoma, 18 pairs adenocarcinoma, 2 pairs adeno-squamous carcinoma) were enrolled in GSE31552 dataset. Genes were differentially expressed by comparison of tumor and paired non-tumor samples. Based on adj.P.Val < 0.05 and |Log fold change| > 2, we detected 334 and 1856 genes which showed differentially expression levels in GSE31552 and GSE18842 dataset respectively. Among these genes, 143 up-regulated genes and 123 down-regulated genes were found in both datasets. According to the 20 highest |Log fold change| in two GES datasets, six genes including MMP12, TPX2, DSG3, SFTPC, TMEM100 and AGER were extracted for further analysis.

### Gene expression analysis

**Fig. 1:**
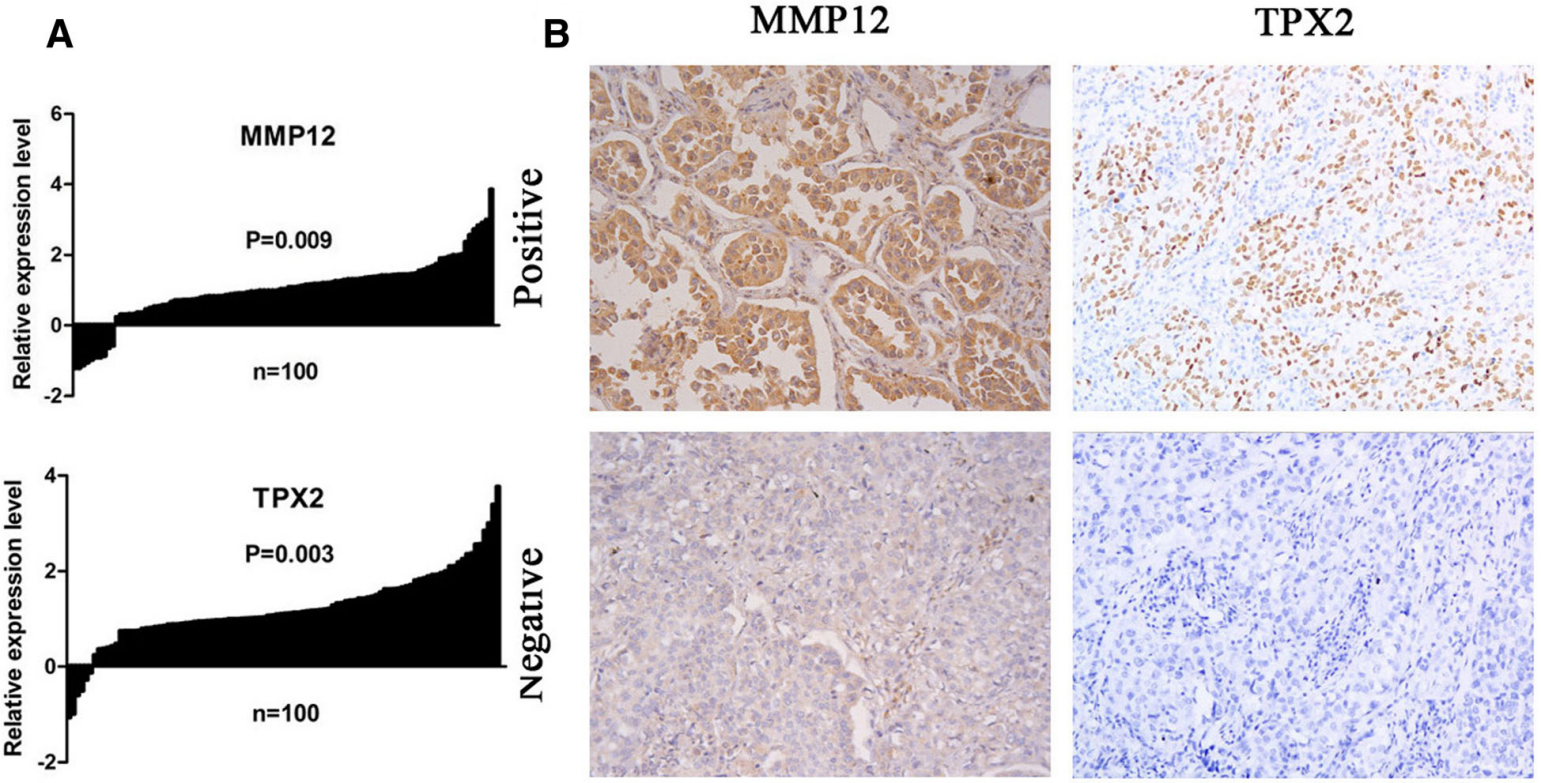
The candidate gene expression in non-small cell lung cancer. **a** Quantitative reverse transcriptase polymerase chain reaction results of two selected genes (TPX2 and MMP12). **b** Representative immunohistochemical staining showing protein expression in the invasive component of tumors (× 200)

Quantitative RT-PCR was carried out to examine whether these six genes were differentially expressed between cancer and normal tissue. The results from 100 tumor and paired normal lung tissue specimens revealed that two of the six genes (TPX2 and MMP12) showed significant expression difference between tumor and normal lung tissue(*P* < 0.05,Fig. 1a). However, there was no significant expression difference in other four genes (DSG3, SFTPC, TMEM100 and AGER) (*P* > 0.05, Additional file 1: Figure S1). As a result, TPX2 and MMP12 genes were selected to perform further analysis.

### Immunohistochemistry for TPX2 and MMP12 expression

The protein expression of TPX2 and MMP12 was examined by immunohistochemistry in 152 tumor samples. In the carcinoma cells, TPX2 staining was mainly found in the nuclei, while MMP12 expression was mainly observed in the cytoplasm of tumor cells. In these samples, the positive expression rates of TPX2 and MMP12 were up to 48.7% (74/152) and 58.6% (89/152), respectively (Fig. 1b).

### The construction of survival prediction model

The median follow-up time for all patients was 31 months (ranged from 3 to 78 months). Univariate Cox analysis showed that TNM stage, tumor grade, postoperative adjuvant therapy, TPX2 expression and MMP12 expression were significantly associated with DFS (*P* < 0.05). Then multivariate Cox proportional hazards regression analysis revealed that TNM stage, tumor grade, TPX2 expression and MMP12 expression were independent predictors (*P* < 0.05, Table 2). Our prognostic model for DFS was calculated as:

**Table 2:** Univariate and multivariate Cox proportional hazards regression for disease-free survival

Variables	Category	Univariate			Multivariate		
		HR	95%CI	*P*-value	HR	95%CI	*P*-value
Age (years)	< 60	1.00					
	≥60	1.81	0.95–3.67	0.089			
Gender	Male	1.00					
	Female	1.04	0.83–1.50	0.884			
Smoking status	Never-smoker	1.00					
	Ever-smoker	1.05	0.79–1.86	0.913			
TNM stage	I	1.00			1.00		
	II	1.35	1.16–2.48	0.002	1.33	1.11–2.45	0.003
	III	2.24	1.39–3.59	<0.001	2.32	1.45–3.68	<0.001
Grade	Well-differentiated	1.00			1.00		
	Moderately-differentiated	1.24	1.10–2.28	0.008	1.27	1.12–2.40	0.005
	Poorly-differentiated	1.81	1.53–2.92	<0.001	1.85	1.55–2.93	<0.001
Histology	Squamous cell carcinoma	1.00					
	Adenocarcinoma	0.97	0.73–1.48	0.241			
Adjuvant therapy	Yes	1.00			1.00		
	No	0.90	0.80–0.96	0.042	0.93	0.82–1.04	0.059
TPX2	Negative	1.00			1.00		
	Positive	1.62	1.21–2.35	<0.001	1.60	1.18–2.31	<0.001
MMP12	Negative	1.00			1.00		
	Positive	1.76	1.32–2.61	<0.001	1.74	1.30–2.59	<0.001

Y = 3.234*TNM + 2.928*Grade + 0.026*TPX2 + 0.025*MMP12.

**Fig. 2:**
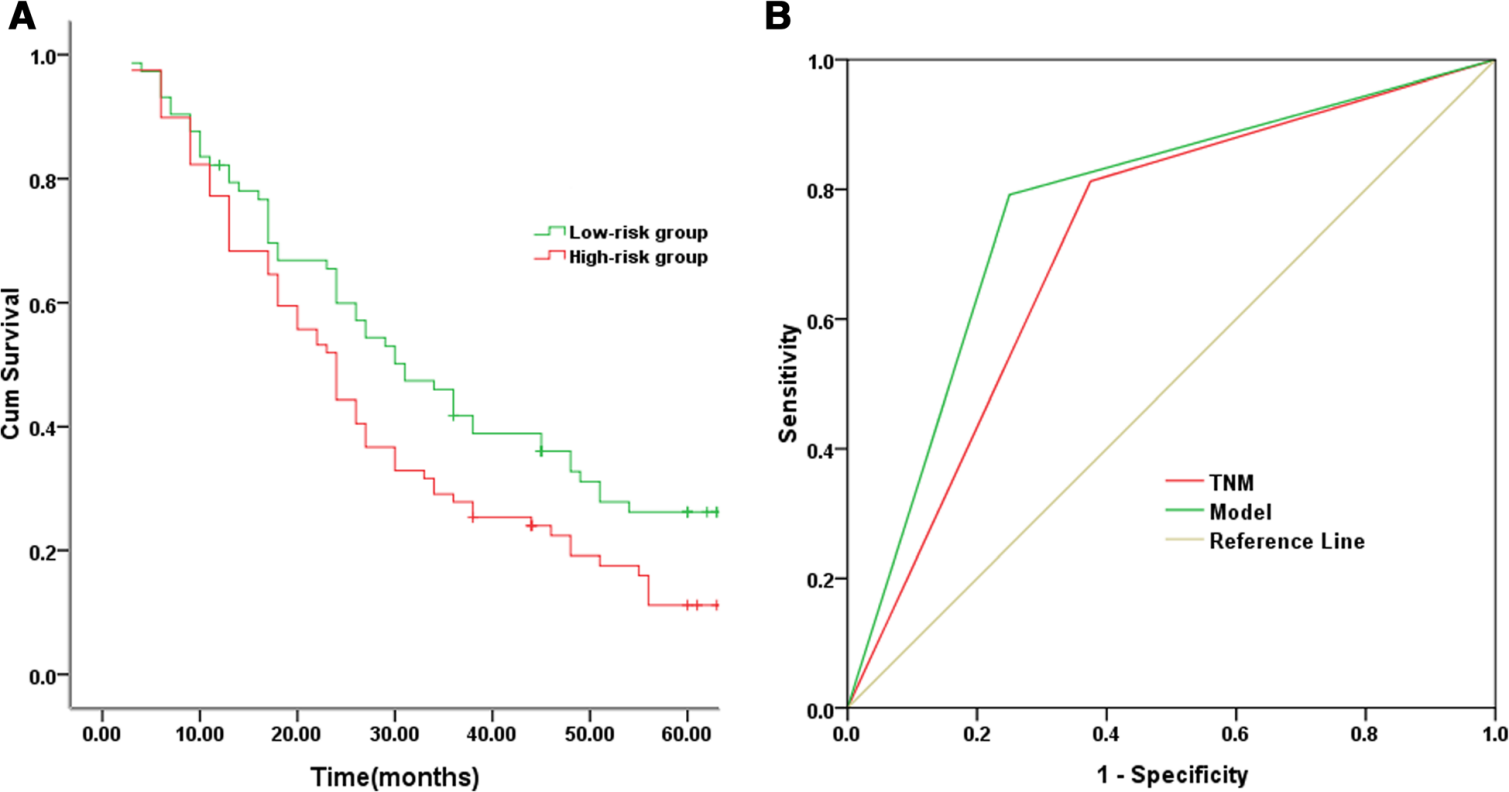
Disease-free survival prediction by prognostic model. **a** Differences in survival between subgroups are assessed by log-rank tests. **b** The predictive ability of the prognostic model as compared with the TNM stage model by ROC curves

Patients were ranked and divided into high-risk group (*n* = 72) or low-risk group (*n* = 80) by using median risk score as the cut-off value. As shown in Fig. 2a, the 5-year DFS rate in high-risk group was significantly lower than that in low-risk group (17.6%vs26.2%, *P* = 0.025). The area under the ROC curve (AUC) value for the survival model was higher than that for TNM system (0.771 (95%CI, 0.689–0.853) vs 0.719 (95%CI, 0.633–0.804)) (Fig. 2b).

As for OS, the results of univariate and multivariate Cox analysis were displayed in Table 3. TNM stage, tumor grade, postoperative adjuvant therapy, TPX2 expression and MMP12 expression were all associated with OS (*P* < 0.05). Further multivariate Cox regression analysis showed that TNM stage, tumor grade, TPX2 expression and MMP12 expression were independent prognostic factors (P < 0.05). The predictive model was calculated as described in the equation:

**Table 3:** Univariate and multivariate Cox proportional hazards regression for overall survival

Variables	Category	Univariate			Multivariate		
		HR	95%CI	*P*-value	HR	95%CI	*P*-value
Age (years)	< 60	1.00					
	≥60	1.25	0.96–3.18	0.105			
Gender	Male	1.00					
	Female	1.05	0.80–1.43	0.849			
Smoking status	Never-smoker	1.00					
	Ever-smoker	1.03	0.84–1.67	0.918			
TNM stage	I	1.00			1.00		
	II	1.30	1.12–2.39	0.003	1.34	1.15–2.46	0.001
	III	2.21	1.34–3.49	<0.001	2.28	1.42–3.56	<0.001
Grade	Well-differentiated	1.00			1.00		
	Moderately-differentiated	1.23	1.90–2.31	0.006	1.31	1.18–2.43	0.004
	Poorly-differentiated	1.80	1.48–2.95	<0.001	1.81	1.47–2.92	<0.001
Histology	Squamous cell carcinoma	1.00					
	Adenocarcinoma	0.91	0.75–1.38	0.239			
Adjuvant therapy	No	1.00			1.00		
	Yes	0.89	0.77–0.94	0.036	0.91	0.81–1.03	0.062
TPX2	Negative	1.00			1.00		
	Positive	1.65	1.21–2.37	<0.001	1.61	1.14–2.26	<0.001
MMP12	Negative	1.00			1.00		
	Positive	1.72	1.30–2.26	<0.001	1.73	1.28–2.54	<0.001

Y = 3.223*TNM + 3.114*Grade + 0.030*TPX2 + 0.025*MMP12.

**Fig. 3:**
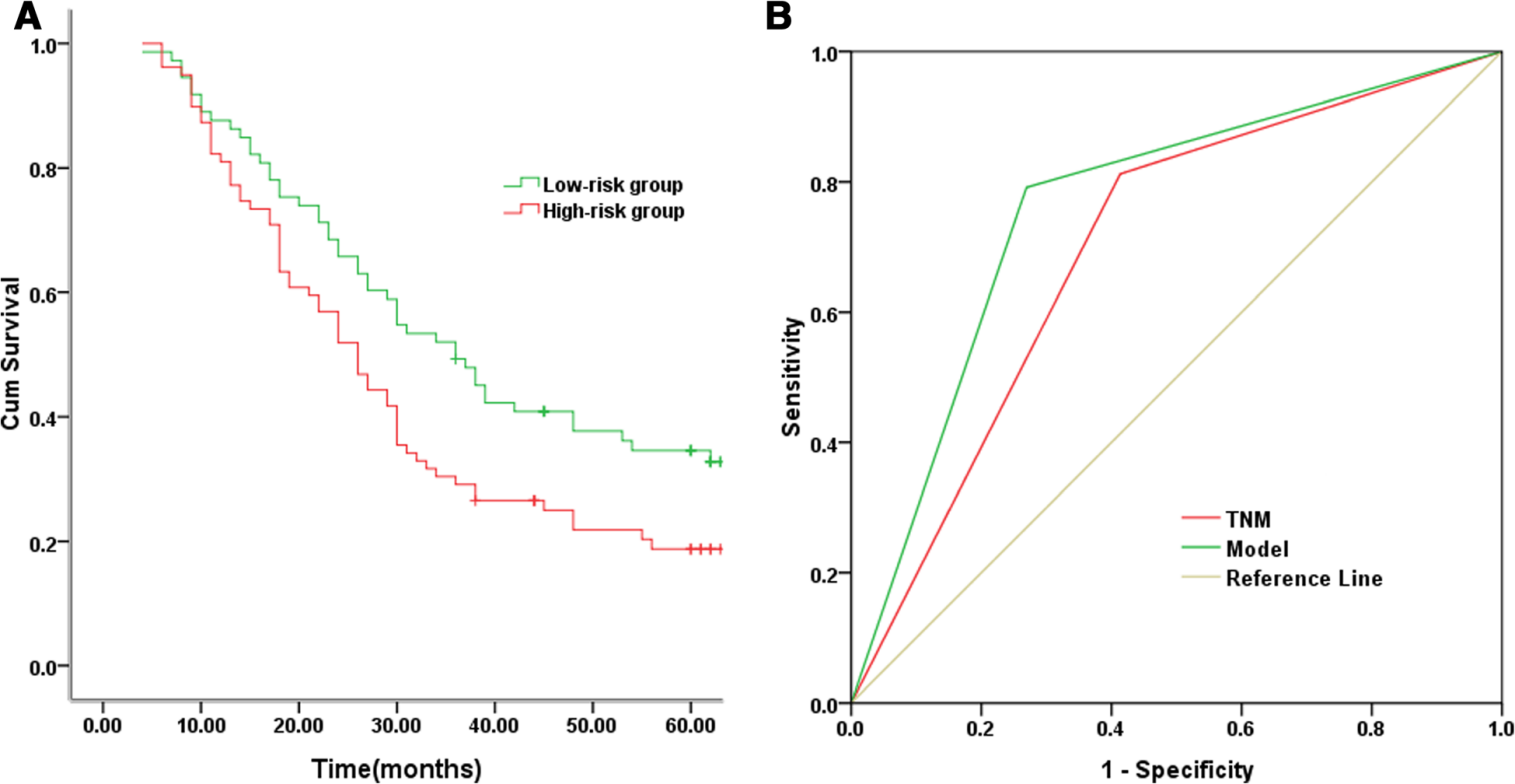
Overall survival prediction by prognostic model. **a** Postoperative survival curves of overall survival based on the survival prediction model by the Kaplan-Meier analysis and log-rank test. **b** The predictive ability of the prognostic model as compared with the TNM stage model by ROC curves

According to the cut-off value, all patients were divided into either high-risk group (*n* = 71) or low-risk group (*n* = 81). Kaplan–Meier curves showed that patients in high-risk group had a worse outcome than those in low-risk group, and the 5-year OS rates were 21.9% and 37.7%, respectively (*P* = 0.021) (Fig. 3a). The AUC value of the survival model was larger than that of TNM stage (0.761 (95%CI, 0.678–0.844) vs 0.700 (95%CI, 0.612–0.787)) (Fig. 3b).

## Discussion

NSCLC clinical outcomes are heterogeneous, and some patients with advanced cancer may have a better prognosis than clinical expectations, and vice versa. At present, the TNM staging system has limited power to predict survival. During the past decades, considerable efforts have been made toward the development of gene-expression-based prognostic biomarkers for NSCLC [14–17]. As a result, well combination of molecular markers and traditional staging system may improve the predictive power. Multivariable analysis in our cohort identified TNM stage and tumor grade as independent prognostic factors (Table 3), which were highly consistent with studies concerning risk factors in NSCLC [18, 19]. In the present study, we developed a predictive survival model containing clinical stage and two molecular biomarkers for NSCLC. Our predictive model provided more prognostic information than TNM staging system alone. It involved molecular factors which may be found to predict survival except for TNM stage. The predictive power of model was higher than that of clinical stage, with the AUC of 0.771 and 0.761. NSCLC patients which were predicted to be high-risk had worse outcome and were more prone to experience tumor recurrence or metastasis. These patients were characterized by higher tumor stage and overexpression of TPX2 and MMP12. However, heterogeneity existed within the stageIIgroup. Some patients with stageIIhad good estimated survival. In fact, they were divided into high-risk group by our predictive model and had bad outcomes.Under-treatment caused by traditional standardized therapy regimens could be avoided. Although the AUC value of our model was slightly larger that of TNM stage, it should be pointed that our model cannot supersede TNM staging and should be used in conjunction with TNM stage. Our predictive survival model hence provides a useful and objective adjunct to current staging criteria that incorporates the heterogeneity existing in the biology of NSCLC. Hence, this needs to be kept in mind when interpreting our result.

MMP12 is one of the metalloproteinases (MMPs), and it causes degradation of the extracellular matrix and basement membranes, and takes part in the pathogenesis of tissue destructive processes in many diseases [20]. Overexpression of MMP12 was observed in various cancers, including gastric cancer, colon cancer and hepatocellular carcinoma [21–23]. And the up-regulation of MMP12 was associated with poor prognosis of cancer [21, 24]. In this study, multivariate Cox regression analysis showed that MMP12 expression was an independent prognostic factor. NSCLC patients with positive expression of MMP12 had a higher Hazard Ratio value for DFS and OS (1.72 (95%CI,1.30–2.26);1.73(95%CI1.28–2.54)). Knockdown of MMP12 inhibited proliferation and invasion of lung adenocarcinoma cells followed by the down-regulation of proliferating cell nuclear antigen (PCNA) and vascular endothelial growth factor (VEGF) [25].These results demonstrated that MMP12 played an important role in tumor invasiveness and metastasis.

In human cells, TPX2 which required for microtubule formation is a microtubule-associated protein. In several types of cancers, the overexpression of TPX2 has been reported [26–29]. Moreover, high TPX2 expression was associated with poor survival in gastric cancer and NSCLC [14, 30]. We also discovered that patients with overexpreeion of TPX2 had a higher Hazard Ratio value for DFS and OS (1.65 (95%CI,1.21–2.37) and 1.61(95%CI1.14–2.26)). Previous studies have revealed that TPX2-siRNA could decrease the viability and proliferation capacity of cancer cell lines [31, 32]. The above observations suggested us that targeted inactivation of TPX2 may have therapeutic benefits.

Previous studies have reported overall-survival predictive model in NSCLC [15, 16]. In our study, not only overall survival but also disease-free survival were considered as end point. The patients who were at high risk of tumor recurrence or metastasis could get maximal benefit from postoperative adjuvant therapy. In addition, IHC is easy to use in clinical pathology laboratories. However, it should be noted that this study is a retrospective study with limited sample size. Because of the retrospective nature of data collection, the established model failed to enroll some important molecular factors (eg. KRAS mutation and EGFR mutation) [33]. As a result, prospective multicenter study needs to be performed before clinical use.

## Conclusion

In conclusion, our study developed a novel model for predicting the survival of NSCLC patients accurately. These findings could be served as an adjunct to the current clinical risk stratification systems. Our methodology may be useful to both patients and clinical doctors in terms of therapeutic strategies.

## Additional files

Additional file 1: **Figure S1.** Quantitative reverse transcriptase polymerase chain reaction results of four selected genes. (JPG 135 kb)

Additional file 2: **Table S1.** qPCR primers used in this study. (DOC 36 kb)

## Abbreviations

AUC: Area under curve
DFS: Disease free survival
EGFR: Epidermal growth
FDR: False discovery rate
GAPDH: Dlyceraldehyde-3-phosphatedehydrogenase
GEO: Gene Expression Omnibus
GSE: Gene series
IHC: Immunohistochemistry
KRAS: Kirsten rat sarcoma viral oncogene
MMP12: Matrix metalloproteinase 12
NSCLC: Non-small cell lung cancer
OS: Overall survival
qRT-PCR: Quantitative reverse transcription polymerase chain reaction
ROC: Receiver operating characteristic curve
TNM: Tumor-node-metastasis
TPX2: Targeting protein for xklp2
