# Supplementary figures and images for: A molecular and staging model predicts survival in patients with resected non-small cell lung cancer
Source: BMC Cancer. 2018 Oct 11;18:966. doi: 10.1186/s12885-018-4881-9 (PMC6180609; doi:10.1186/s12885-018-4881-9)

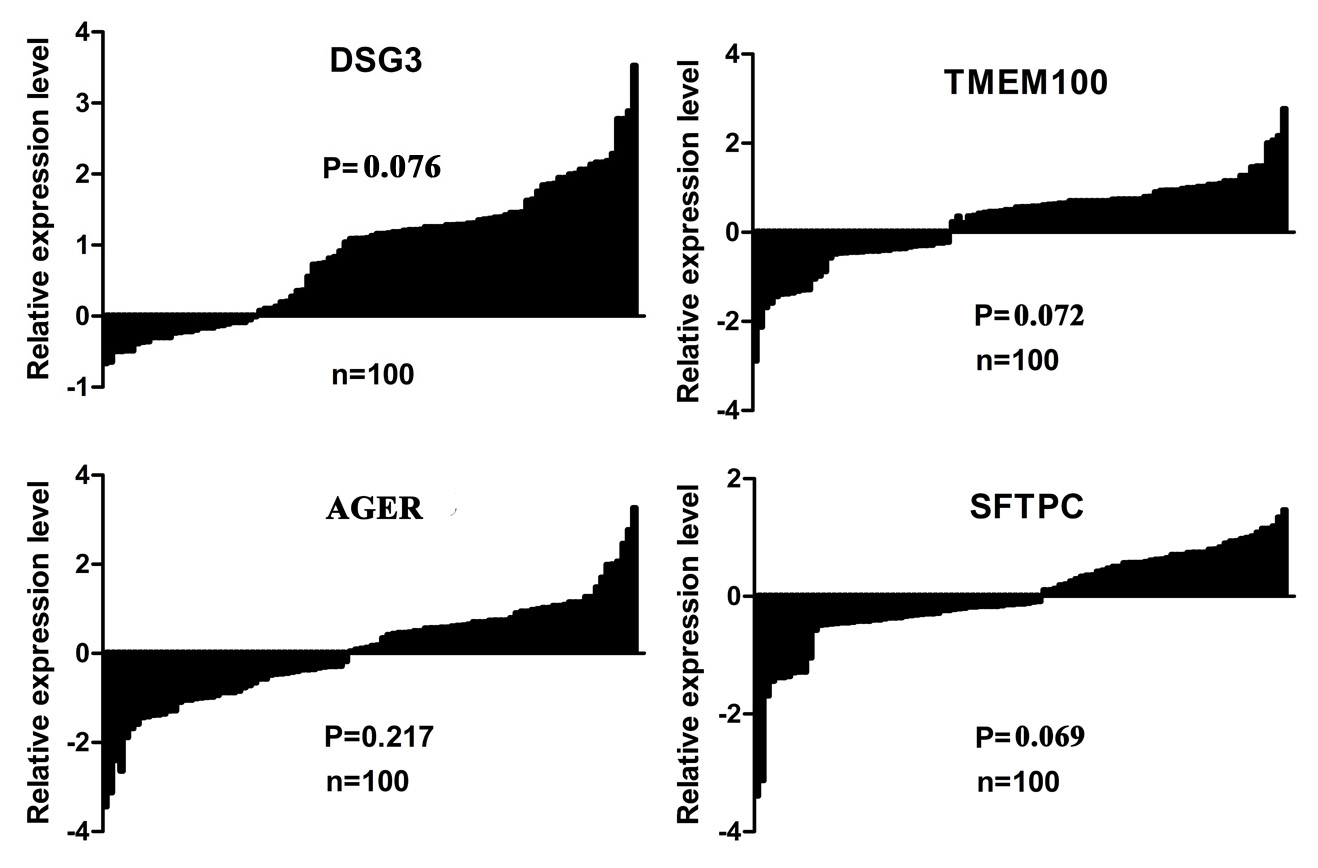

Supplement: Supplementary file 1 — Figure S1. Quantitative reverse transcriptase polymerase chain reaction results of four selected genes. (JPG 135 kb) [file 12885_2018_4881_MOESM1_ESM.jpg]
