# Supplementary material for: A molecular and staging model predicts survival in patients with resected non-small cell lung cancer
Source: BMC Cancer. 2018 Oct 11;18:966. doi: 10.1186/s12885-018-4881-9 (PMC6180609; doi:10.1186/s12885-018-4881-9)
Supplement: Supplementary file 2 — Table S1. qPCR primers used in this study. (DOC 36 kb) [file 12885_2018_4881_MOESM2_ESM.doc]

**Table S1. qPCR primers used in this study.**

| **Gene** | **Primer sequences (5' -> 3')** | Amplicon size |
| --- | --- | --- |
| MMP12 | F’- GGAATCCTAGCCCATGCTTTT | 174bp |
|  | R’- CATTACGGCCTTTGGATCACT |  |
| TPX2 | F’- ATGGAACTGGAGGGCTTTTTC | 93bp |
|  | R’- TGTTGTCAACTGGTTTCAAAGGT |  |
| DSG3 | F’- GCAAAAACGTGAATGGGTGAAA | 130bp |
|  | R’- TCCAGAGATTCGGTAGGTGATT |  |
| SFTPC | F’- CACCTGAAACGCCTTCTTATCG | 96bp |
|  | R’- TTTCTGGCTCATGTGGAGACC |  |
| TMEM100 | F’- TGCTGTGGTTGTCTTCATCG | 183bp |
|  | R’- CTCTCCCGTCTCTTGGCTTTC |  |
| AGER | F’- ACTACCGAGTCCGTGTCTACC | 79bp |
|  | R’- GGAACACCAGCCGTGAGTT |  |
| GAPDH | F’- GACATCAAGAAGGTGGTGAA | 155bp |
|  | R’- TGTCATACCAGGAAATGAGC |  |
